# Targeting microbial pathogenic mechanisms as a novel therapeutic strategy in IBD

**DOI:** 10.1186/s10020-024-00840-9

**Published:** 2024-08-13

**Authors:** Paul F. Miller

**Affiliations:** Lighthouse Biopharma Consulting, LLC, 39 Emerald Glen Lane, Salem, CT 06420 USA

**Keywords:** Pathobiont, Dysbiosis, Pathogenesis, IBD, Microbiota, Mycobiota

## Abstract

**Background:**

Current therapy for patients suffering from inflammatory bowel diseases (IBD) is focused on inflammatory mechanisms exclusively and not the dysbiotic microbiota, despite growing evidence implicating a role for intestinal microbes in disease.

**Main body:**

Ongoing research into the intestinal microbiota of IBD patients, using new technologies and/or deeper application of existing ones, has identified a number of microorganisms whose properties and behaviors warrant consideration as causative factors in disease. Such studies have implicated both bacteria and fungi in the pathogenesis of disease. Some of these organisms manifest mechanisms that should be amenable to therapeutic intervention via either conventional or novel drug discovery platforms. Of particular note is a deeper characterization of microbial derived proteases and their destructive potential.

**Conclusion:**

Given the steady progress on the mechanistic role of the microbiota in inflammatory diseases, it is reasonable to anticipate a future in which therapeutics targeting microbial derived pathogenic factors play an important role in improving the lives of IBD patients.

## Background

It is now well-accepted that the host’s intestinal microbiota is a contributing factor in inflammatory bowel diseases (IBD). Changes in the diversity and functionality of the gut microbial community in IBD patients compared with healthy individuals, a situation referred to as dysbiosis, has been noted for many years (Ott et al. 2004, Tamboli et al. 2004). Further defined in disease association studies as a reduction in microbial diversity (Frank et al. 2007; Sartor 2008), subsequent work has sought to define mechanisms that explain how these changes contribute to a patient’s disease status and whether they are simply correlative or indeed causative (Gevers et al. 2014). This distinction has important therapeutic consequences, as current IBD therapies are almost exclusively focused on the dysregulated immune system that is a hallmark of disease. Logically, immune suppressive agents have been a mainstay in disease treatment, beginning with broadly acting, non-specific drugs such as steroids and evolving to include newer therapeutics that target specific pathogenic inflammatory factors. This latter category includes the biologics that neutralize or sequester pro-inflammatory cytokines or trafficking molecules that direct immune cells to the intestinal epithelium. Unfortunately, these agents typically show efficacy in inducing clinical remission in less than half of the studied patients in pivotal clinical trials, with the responder population challenging to identify prospectively. For example, Paramsothy and colleagues reviewed registration trial data for new agents in Crohn’s disease (CD) and ulcerative colitis (UC) patients, and the summary tables therein are informative (Paramsothy et al. 2018). Surgery, while delayed, remains an eventual outcome for many patients (Bernstein et al. 2012, Oresland and Faerden 2015).

Targeting inflammation, while effective in many patients, may not address mechanisms operating at the initial phases of disease onset. Interestingly, while a large number of host susceptibility genes have been associated with IBD, several are involved in the recognition of, or response to, microbes or microbial products (Venema et al. 2017). Given the large number of bacterial species in an individual, along with the heterogeneous composition of microbial communities across patients, the prospect of identifying individual causative organisms is daunting. Nonetheless, the field of microbiome research has advanced steadily over the past 15 years, progressing from its initial phase of microbial cataloging to mechanistic characterization at the genetic and metabolic level (Britton and Faith 2021). The initial descriptive community membership studies, including those comparing healthy individuals with patients burdened by varying diseases, may have had the unintended effect of shifting focus away from individual species of bacteria since obvious pathogenic fingerprints didn’t immediately emerge. One cause of this situation was the use of 16S sequencing to characterize microbial community composition. While quite useful and state-of-the art at the time, these methods mostly identify organisms at the family or genus level, and rarely as species. Importantly, bacterial species consist of numerous strains that can be distinguished by the presence, absence or reorganization of distinct genes that alter the functions of the organism in different environments. Consequently, the use of deep sequencing methods that allow strain-level characterization of organisms, particularly when coupled with culturing of these organisms from the same patients, has enabled the implication of specific pathobionts in disease and merited their further investigation as therapeutic targets.

To further assist in bridging the gap between organism cataloging and the mechanistic implication of specific microbes, new technologies have been developed to identify those strains that can induce disease-relevant effects in pre-clinical models, beginning with patient-derived biosamples (Palm et al. 2014; Geva-Zatorsky et al. 2017; Britton et al. 2020). These tools, and several others mentioned below, offer insights into the interface between the intestinal microbiota and the host’s immune system, and have provided additional means to explore the role of specific microbes as initiators or exacerbators of disease. This review will highlight several examples of these associations, with a focus on those organisms where a greater burden of evidence exists, including identified mechanisms of pathogenesis.

## Main text

*Supporting evidence for a causative role of the microbiome in IBD* Before proceeding to a discussion of individual organisms, it is perhaps useful to briefly summarize other related treatment approaches that support a causal role for the microbiota in IBD. Antibiotic treatment is one such paradigm, and has been advocated as a means to alter the composition of a dysbiotic microbial community and reduce the numbers of presumed problematic pathogens, including enteric Gram-negative species such as *E. coli*. It is hoped that such treatment would also provide an opportunity for beneficial organisms to expand and restore a healthier community structure. Concerns with this approach include side effects of antibiotic use, an increased risk of *C. difficile* infection, a mismatch between the antibiotic regimen applied and the actual offending organisms in a given patient, and the emergence of antibiotic resistance (Nitzan et al. 2016). It is challenging to summarize the overall benefit of antibiotic therapy in the treatment of IBD, as many studies are small in size and use different drug regimens and treatment courses. Nitzan and colleagues summarized available data from antibiotic treatment studies in CD and UC patients, noting that a greater amount of data was available for CD than for UC. In CD patients, there was a trend towards a benefit of antibiotic treatment relative to placebo, with metronidazole showing improved performance compared with ciprofloxacin in inducing clinical remission. Results from trials involving UC patients were more mixed, but overall, the analyses have shown an increased likelihood of achieving clinical remission in antibiotic treated cohorts (Nitzan et al. 2016). Thus, while the side effects and undesirable consequences associated with antibiotic treatment limit its usage, the benefits are consistent with an active role of the microbiota in IBD.

Following the same logic, correction of dysbiosis in IBD patients as an approach to disease treatment could be accomplished through fecal microbiota transplantation (FMT), which involves the instillation of intestinal bacteria in the form of a stool sample from a healthy donor to a recipient subject. This method has proven successful in the prevention of recurrent *Clostridioides difficile* infection, motivating its exploration in IBD. Imdad and colleagues reviewed a small number of placebo-controlled FMT studies involving UC patients, and concluded that while the procedure appeared to produce a roughly twofold increase in remission rates, the studies were too small to draw clear conclusions (Imdad et al. 2018). Similarly, Fehily reviewed fifteen published FMT studies in CD patients with encouraging preliminary results. However, the authors suggest the need for large, controlled studies to solidify these findings (Fehily et al. 2021). More recently, Feng and colleagues scrutinized published trials of FMT for the treatment of UC. After focusing on 13 studies with the highest data quality, they concluded that FMT has a clear benefit for UC patients in inducing clinical and endoscopic remission (Feng et al. 2023).

Against this backdrop, an alternative to the restoration of a “healthy microbiota” is the identification of problematic organisms in patients’ dysbiotic communities, and developing therapeutic approaches based on the pathogenic mechanisms elaborated by these species. Such an approach is not without its challenges, including the translational validity of rodent models in replicating human disease as well as the technical hurdle of targeting individual organisms without affecting other benign or beneficial members of the ecosystem. Indeed, the selective removal of individual species or strains from a complex community is a daunting challenge. Currently available antibiotics would affect additional bystander organisms beyond the targeted pathogenic strains, as mentioned above. Accordingly, bacteriophages, or phages, which are viruses that infect specific species of bacteria, have drawn attention as potential therapeutics that could be used to precisely remove organisms of interest in a targeted fashion. The intestinal microbiota is in actuality a milieu of both microbes and their infecting phages, and consequently stool samples or human waste streams can be sampled for viruses capable of killing a bacterium of interest. As bacterial resistance to infection by a specific phage can emerge quickly, therapeutic approaches include cocktails of these viruses as well as engineered versions to increase bacterial killing (Hsu et al. 2019, Kilcher and Loasner 2019). While conceptually intriguing and promising, this approach is still under development. Finally, numerous animal models exist to support the development of drug discovery projects (Kiesler et al. 2015). Different models have strengths and weaknesses in supporting IBD research, and it is unclear as to which of these will be the most translationally useful in supporting the development of microbiome-targeted therapeutics. Nonetheless, substantial progress has been made in identifying candidate causative organisms in IBD as well as in progressing novel therapeutics against these, as is discussed in the following sections.

*Adherent-invasive* *Escherichia coli* (AIEC) Numerous studies have identified an increase in the abundance of Enterobacteriaciae in the intestinal microbiota of IBD patients, including *E. coli*. A subset of *E. coli* strains was subsequently implicated in disease due to their association with, and invasion of, the intestinal epithelium. Originally implicated in IBD over 25 years ago (Darfeuille-Michaud et al. 1998), the identification of these strains remains challenging due to an absence of specific virulence factors or genetically distinguishing markers (Nash et al. 2010). Consequently, the identification of AIEC strains has been based on in vitro, cell-based assessments including the invasion and replication in J774 mouse macrophages as well as human epithelial Caco2 cells (Glasser et al. 2001, Boudeau et al. 1999). More recently, Kittana et al. reported on the evaluation of a more comprehensive set of AIEC isolates to assess the utility of in vitro assays (Kittana et al. 2023). These investigators systematically analyzed a collection of intestinally derived *E. coli* strains from patients and examined the relationship between in vitro and in vivo phenotypes. The ability of strains to survive and replicate in J774 macrophages and Caco2 cells in vitro was most predictive of induction of inflammation in vivo. Consistent with previous observations, however, the confirmed AIEC isolates were not associated with specific distinguishing genetic markers or known pathogenicity factors. The authors conclude that AIEC character is consistent with the adaptive behavior of *E. coli* as a species, with environmental factors selecting for favorable combinations of genes that support growth in an inflammatory environment, and without the emergence of specific, definable clones.

Interest in AIEC as a potential driver of disease in a subset of IBD patients has motivated the search for targeted therapeutics. One approach is the development of a bacteriophage cocktail that specifically targets these organisms. Titecat and colleagues recently described the pre-clinical safety and efficacy of a seven-phage combination therapy, called EcoActive, in a DSS mouse model of intestinal inflammation (Titecat et al. 2022). Using a collection of 210 AIEC strains from the US and Europe, this cocktail was found to be effective in lysing 95% of these isolates in vitro. High dose treatment of DSS-treated mice colonized with the AIEC strain LF82SK for 15 days resulted in a significant decrease in bacterial counts along with a decrease in intestinal inflammatory measures. Intralytix, a co-discoverer of the EcoActive phage cocktail, announced in August 2023 that a Phase 1/2a human clinical trial had been initiated to explore the safety and efficacy of the therapeutic in patients with inactive Crohn’s disease (Intralytix company website).

Taking a different approach, investigators at the biotech company Enterome have identified a novel, oral, small molecule therapeutic that targets the bacterial adhesion protein FimH, used by AIEC to bind to epithelial cells. The compound, called sibofimloc (subsequently licensed to Takeda Pharmaceuticals as TAK-018), blocked the ability of AIEC to adhere to human intestinal explants and induce inflammation (Chevalier et al. 2021). The company successfully completed a Phase 1b study in patients with active Crohn’s disease, demonstrating that sibofimloc was safe and well tolerated. A Phase 2 trial in post-operative Crohn’s disease patients was announced in late 2021; however, in early 2023 Takeda communicated in a corporate portfolio update that the clinical program had been discontinued due to enrollment challenges. Consequently, the clinical hypothesis behind the FimH blocking approach remains unanswered.

*Klebsiella pneumoniae* This member of the Enterobacteriaciae has attracted attention more recently as a candidate pathobiont in IBD. A high-powered group of microbiome researchers used gnotobiotic mouse models to investigate the role of oral bacteria in the induction of intestinal inflammation following ectopic colonization at that site (Atarashi et al. 2017). Previous studies had identified an increased presence of oral microbes in the gut microbiota of IBD patients (Gevers et al. 2014), motivating the search for individual organisms that may be playing proinflammatory roles. Characterizing the intestinal microbiota from mice colonized by salivary bacteria from an IBD patient that had developed intestinal inflammation, *K. pneumoniae* was identified as a key driver of colitis. A related species, *K. aeromobilis*, also induced inflammation in susceptible mouse models. *Klebsiella* species were also found to be enriched in alcoholic patients as well as individuals suffering from GERD and PSC. Regarding the latter disease, Nakamoto and colleagues demonstrated that the microbiota from PSC patients could induce a TH17 response in the liver of gnotobiotic mice, and that *K. pneumoniae* from these communities promoted epithelial damage and was associated with bacterial translocation in preclinical models (Nakamoto et al. 2019).

Similar to AIEC, a therapeutic bacteriophage approach is being advanced by the biotech company BiomX as a candidate treatment for this organism. This therapeutic cocktail stemmed from a collaborative investigation on the colitigenic potential of *K. pneumoniae* strains from IBD patients that extended the findings of Atarashi (Federici et al. 2022). An assemblage of 5 phages was identified that could suppress intestinal inflammation in susceptible mice colonized with these strains. A healthy volunteer study demonstrated safety and tolerability of the phage cocktail, as well as the successful transit of the orally administered therapeutic to the lower GI tract. However, this candidate therapeutic is not currently listed in Biomx’ active portfolio, and its status is unclear. In a similar fashion, Ichikawa and colleagues developed a phage cocktail that was effective in suppressing hepatobiliary injury in *K. pneumoniae* colonized mice (Ichikawa et al. 2023).

*Enterotoxigenic Bacteroides fragilis* (ETBF) Among the better studied organisms highlighted in this review, strains of *B. fragilis* bearing the *bft* enterotoxin gene have emerged in several different pathogenic contexts including diarrheal disease, inflammatory bowel disease and colorectal cancer (Sears 2009; Valguarana 2019). This organism is able to induce a persistent colitis in standard C57BL/6 mice, making it somewhat unique among pathobionts (Rhee et al. 2009). The toxin stimulates the disruption of epithelial barriers with the concomitant release of fragments of the adherens junction protein E-cadherin (Wu et al. 2007) although E-cadherin is not the direct target. The association of this bacterial strain with IBD underscores the importance of interrogating the microbiota at greater depth than 16S sequencing, whose resolution is limited to the family or genus level. Specifically, *B. fragilis* detection in stool samples is generally no cause for alarm; in fact, this species has been noted to have anti-inflammatory properties, due in part to its capsule composition (Mazmanian et al. 2005). In stark contrast, ETBF strains containing the *bft* gene can be identified only by deeper sequencing methods or specific PCR detection of the toxin gene, the presence of which is sufficient to convert a common commensal into a true pathobiont (Sears 2009).

Bft is a secreted metalloprotease that is required for the pathogenic properties associated with ETBF. It is synthesized as a pro-protein that is cleaved enzymatically to release the mature active protease. Sears and colleagues have identified pro-inflammatory and pro-oncogenic pathways induced in epithelial cells as a consequence of Bft binding. As a metalloenzyme, the prospects for identification and progression of specific inhibitors as drug candidates seems promising, and among the organisms of interest in this review, the toolkit of target crystal structure (Goulas et al. 2011), enzyme assays, cellular assays and animal models could support drug discovery efforts. Metz and colleagues described the identification of chenodeoxycholic acid as a candidate therapeutic for ETBF-mediated disease (Metz et al. 2019). The molecule was shown to mitigate the cellular effects of Bft exposure through direct binding to the toxin, although an effect on protease activity was not demonstrated. Artizan Biosciences, a biotechnology company founded based on the IgA-Seq technology from the laboratory of Richard Flavell (Palm et al. 2014), has also reported on a small molecule inhibitor of the Bft protease that it is progressing towards human clinical trials (Miller 2022).

*Other Bacteroides species* Utilizing a sophisticated computational approach, Mills and colleagues identified the abundance of Bacteroides-derived proteases as highly correlated with disease status in ulcerative colitis patient stool samples using an integrated multi-omics data approach (Mills et al. 2022). *B. vulgatus* proteases showed the highest correlation, with *B. dorei* also implicated. *B. vulgatus* increased epithelial barrier permeability in vitro and induced colitis in a mono-colonized IL-10 deficient mouse model; both features were reversed by administration of a broad-spectrum protease inhibitor cocktail. Intriguingly, bacterial-derived protease activity correlated with disease severity in stool samples from this patient cohort, and high protease samples conferred higher disease scores in recipient germ free mice as compared with low protease samples obtained from patients with quiescent disease. These findings further highlight the potential role of microbial-derived proteases in IBD, as well as the power of the multi-omics strategy.

*Enterococcus faecalis* Among the organisms of interest as pathobionts in IBD, *E. faecalis* satisfies many criteria that qualify it for suspicion. Common commensals in many humans, the enterococci have evolved over time, notably in the context of the antibiotic era (Gilmore et al. 2013). The streamlining of these organisms’ genomes has included retention of mechanisms that permit epithelial colonization and translocation. In the context of IBD, Steck and colleagues showed that *E. faecalis* is able to induce colitis in the mouse IL-10^−/−^ model, in a manner that requires expression of the GelE gelatinase (Steck et al. 2011). This secreted metalloprotease has been implicated in the ability of *E. faecalis* to translocate across the intestinal epithelial barrier, and the purified enzyme increases epithelial barrier permeability in cell-based assays. Clinically, increased levels of *E. faecalis* were observed in IBD patients compared with healthy controls (Zhou 2016). However, GelE is produced in only a subset of *E. faecalis* strains (Galloway-Pena et al. 2011), and the presence of the *gelE* gene was not determined in the Zhou study. Related to the IBD observations with this organism, extensive work from John Alverdy and colleagues has shown that *E. faecalis* colonizes surgical anastomosis sites in patients experiencing leakage and failure of the resection. IBD patients are often the subjects of these procedures, and a role for the organism in surgical site failure was replicated in a rat intestinal resection model. Notably, the *gelE* gene was required for anastomotic failure in this model, and the mechanism involved activation of the tissue matrix metalloproteinase MMP9 (Shogan et al. 2015).

With respect to therapeutic opportunities, the GelE metalloprotease represents a potentially druggable target, and Steck showed that GelE protease activity as well as the ability of the enzyme to disrupt epithelial barriers could be blocked by marimastat, a broadly acting MMP inhibitor (Steck et al. 2011). These results suggest that a drug discovery effort focused on developing selective inhibitors of the bacterial enzyme may be fruitful. Alternatively, work conducted by Schnabl and colleagues showed that bacteriophage therapy targeting *Enterococcus faecalis* was effective in a mouse model of alcoholic hepatitis that was induced by this organism (Duan et al. 2019). More recently, Iida and colleagues demonstrated a putative role for *gelE-*positive *E. faecalis* in the induction of liver carcinogenesis (Iida et al. 2011). Thus, this organism appears to utilize a secreted metalloprotease in the induction of intestinal and adjacent liver diseases.

*Clostridium perfringens* More commonly recognized as a pathogenic cause of food-borne illness and gas gangrene, this organism has also been implicated in IBD, albeit with less literature and clinical evidence. *C. perfringens* elaborates a variety of toxins, the presence and combination of which underlie strain typing (Kiu and Hall 2018). In an examination of gut microbial sources of gelatinolytic activity, Pruteanu and colleagues identified *C. perfringens* as the most commonly isolated producer of this protease type (Pruteanu et al. 2011). Cultured isolates contained the gene encoding the secreted metalloprotease ColA, and supernatants from these cultured strains were able to degrade the major basement membrane component type IV collagen, and also disrupted rat intestinal barrier function ex vivo using an Ussing chamber system. Subsequent work from this laboratory showed that culture supernatants could decrease the amounts of the tight junction proteins occludin and JAM-1, as well as the adherens junction protein E-cadherin. This activity appears to result from the action of both the metalloprotease ColA and the cysteine protease clostripain which were present in the culture supernatant preparations (Pruteanu and Shanahan 2013). Intriguingly, *C. perfringens* was among the highly IgA-coated organisms identified in IBD patient stool samples by Palm and colleagues (Palm et al. 2014). Consequently, and given its rapid growth rate under permissive anaerobic conditions, it is tempting to speculate that a bloom of this organism in the dysbiotic microbiota of certain IBD patients could contribute to disease. As a therapeutic target, the above work from Pruteanu and colleagues showed that the epithelial disruptive activities associated with *C. perfringens* supernatants could be inhibited by the non-specific metalloprotease inhibitor EDTA. Accordingly, it should be possible to develop optimized inhibitors of the ColA enzyme as potential drug candidates, should confidence in the causative role of this organism in disease increase. Notably, and distinct from the other metalloprotease-producing organisms discussed here, it appears that the *colA* gene is a general feature of *C. perfringens* as a species and is conserved among all isolates studied in this context.

*Candida albicans* While long appreciated as a fungal opportunistic pathogen, interest in *C. albicans* as a causative agent of IBD has increased of late. Changes in the fungal component of the intestinal microbial ecosystem in the context of IBD have been noted, with increased abundance of specific species, including *C. albicans* (Sokol et al. 2017). This organism readily colonizes mucosal surfaces and can activate pro-inflammatory signaling pathways known to be induced in IBD, most notably that of IL-17 (reviewed in Ho et al. 2020). A critical mechanistic breakthrough emerged with the discovery of the virulence factor candidalysin, a 31 amino acid peptide secreted from *C. albicans* hyphae and responsible for cellular damage induction and cytokine production (Moyes et al. 2016). The candidalysin peptide is a cleavage product from a polyprotein encoded by the *ECE1* gene, and mutants that are either lacking *ECE1* or unable to cleave the larger ECE1p protein to release the active candidalysin peptide are defective in pathogenesis (reviewed in Naglik et al. 2019). More recently, Li et al. developed a robust fungal analysis platform utilizing IBD patient-derived samples coupled with in vitro and in vivo models to demonstrate that highly pathogenic *C. albicans* strains capable of damaging immune cells and stimulating inflammatory immune responses could be isolated from the mucosa of ulcerative colitis patients (Li et al. 2022). These effects were shown to be dependent on candidalysin production. Accordingly, *C. albicans* and candidalysin bear strong consideration as potential therapeutic targets for the treatment of IBD.

### Future perspectives

While not inclusive of all organisms that have garnered some measure of suspicion in the pathogenesis of IBD, the information summarized in this review is intended to shed light on a subset of strains and species where the burden of evidence is stronger. An important concept in considering individual microbes in the pathogenic process of IBD is the conditional nature of this role. From a traditional infectious diseases perspective, our microbial denizens have been distinguished as either commensals or pathogens, with an intermediate label of “opportunistic pathogens” afforded to those disease-causing organisms that are most often problematic only in individuals with underlying disease or immune insufficiency. A similar term, “pathobiont”, has emerged to describe normally benign organisms that appear to be pathogenic in IBD patients (Chow et al. 2011) or other chronic diseases (Fine et al. 2020). This can be a challenging concept, as it is unclear why such organisms would be pathogenic in one individual but seemingly innocuous in another. Host genetics is likely one factor that explains this difference.

At least 200 genetic loci have been identified that affect the susceptibility to developing IBD (Liu 2016). Notably, a number of susceptibility genes encode functions associated with the recognition of microbial products, such as NOD2, or in the response to microbial incursion, exemplified by the autophagy related gene ATG16L1 (Venema et al. 2017). As such, one simple explanation for the sensitivity of IBD patients to the presence of pathobionts might be that epithelial barrier disruption by these organisms results in an influx of luminal microbes that triggers a dysregulated inflammatory response due to defects in the mechanisms for sensing or clearing the initial invaders. In addition, and as noted above, the abundance of several of the pathobionts highlighted here is much higher in IBD patients than in healthy carriers. It will be important to genetically characterize IBD patients who are harboring these organisms to determine if genetic factors are associated in organism-specific ways.

The induction of disease in animal models following the introduction of these organisms, along with their increased abundance and ability to disrupt epithelial barriers, is suggestive of a role in the initiation of disease. In a clinical context, assessment of maintenance of remission is an attractive efficacy measure to include in the design of patient trials. This might merit combination studies with standard of care therapy, including biologics, to determine if effective resolution of symptoms and increased time to relapse is observed by blocking both barrier disruption by pathobionts as well as dysregulated inflammation. Since several of the pathogenic factors described here are able to disrupt epithelial barriers, an increased rate of intestinal wound healing may be a benefit of blocking these virulence mechanisms. Strategies for the use of these therapies in the prevention of flares should also be explored.

The ability to identify most, if not all, of the above organisms through the use of companion diagnostic tools would create an opportunity for personalized medicine in the treatment of IBD. Examples of these technologies would include PCR assays for pathogenesis genes (e.g. *bft* in *B. fragilis*) and ELISA assays for the pathogenic factors themselves (e.g. *B. vulgatus* proteases). These could be applied to patient stool samples and would support enrollment decisions in clinical trial recruiting as well as in patient treatment paradigms if specific therapeutics were successfully commercialized. More comprehensively, metagenomic sequencing of patient stool samples could also be used to create a more complete picture of a patient’s intestinal microbiota and the community contexts in which these organisms are found. This characterization would also be valuable in following patients longitudinally to understand the impact of blocking a pathogenicity factor on the persistence and abundance of an organism.

In summary, deeper investigations into the patient-specific behaviors of individual organisms have allowed a greater understanding of the mechanisms by which intestinal microbes can alter the epithelial environment to affect disease. Of particular note is the theme of microbial-derived intestinal proteases, the overall burden of which is higher in IBD patients than healthy individuals. Several disease-associated organisms highlighted here produce these enzymes which are mechanistically linked to their ability to cause disease in relevant models. As evidence continues to build, it is anticipated that drug developers will clinically test the microbial driver hypothesis with new therapeutics, and as a consequence bring a new level efficacy and personalized medicine to IBD patients that is complimentary to existing therapeutic approaches.

## Data Availability

Not applicable.
